# Prescribing patterns of echinocandins in adult patients in a private hospital in Gauteng, South Africa

**DOI:** 10.4102/sajid.v38i1.470

**Published:** 2023-03-14

**Authors:** Anja Grey, Rianda Joubert, Stephan Steyn, Marlene Julyan

**Affiliations:** 1 Department of Medicine Usage in South Africa, Faculty of Health Sciences, North-West University, Potchefstroom, South Africa; 2 Centre of Excellence for Pharmaceutical Sciences, Faculty of Health Sciences, North-West University, Potchefstroom, South Africa

**Keywords:** echinocandins, candidemia, loading dose, daily dose, appropriate duration

## Abstract

**Background:**

Worldwide, the leading cause of invasive candidiasis and the fourth leading cause of hospital-acquired infections are the Candida species (spp.) group. One of the most important tools in fighting such drug-resistant fungi is the appropriate use of antifungal agents.

**Objectives:**

The study aimed to determine echinocandins’ general prescribing patterns and how they are associated with the treatment period.

**Method:**

A quantitative, observational, and descriptive was used, and included patients receiving antifungal treatment in a private hospital in Gauteng, South Africa between 01 January 2015 to 31 December 2015.

**Results:**

Of the 146 patient files included, 102 patients (69.9%) received caspofungin and 44 patients (30.1%) were treated with anidulafungin. For the former, 99 (97.1%) patients received a loading dose (LD) of 70 mg, while 200 mg anidulafungin was only prescribed to 30 patients (68.2%). In line with maintenance dose guidelines, the majority (98.1%) of caspofungin-treated patients received 50 mg IV daily, whereas 4 (3.9%) patients were treated at higher doses (70 mg daily). Anidulafungin was administered at various maintenance doses, including 400 mg (2.3% of patients), 200 mg (52.3%), 100 mg (43.2%) and 50 mg (2.3%) IV daily.

**Conclusion:**

Our results can be utilised to produce a hospital-specific algorithm in terms of Candida-infected patients.

**Contribution:**

These findings contribute to our understanding of prescribing patterns of antifungal agents and the impact thereof on treating *Candida* spp. Infections.

## Introduction

*Candida* species are the main source of invasive candidiasis globally; they are the fourth leading cause of infections acquired within hospital settings. In addition, they are associated with dramatic mortality and morbidity rates.^1^ According to recent reports, while the leading fungal threat to public health^2^ remains *Candida albicans* resistance, the number of patients infected with non-*albicans Candida* spp. is steadily growing. These species include *Candida glabrata, Candida parapsilosis, Candida krusei* and *Candida tropicalis*,^1,3,4,5,6^ which potentially contribute to the increased drug resistance to commonly prescribed pharmacological antifungal drugs. As such, it is widely recognised that the epidemiology-related changes of the *Candida* spp. contribute significantly to antifungal drug resistance.

While research acknowledges that the correct use of antifungal agents is one of the most important factors contributing to drug resistance, inappropriate antifungal use results in fungal species’ resistance developing over time. Negative usage factors include sub-therapeutic dosing and inappropriate or ineffective treatment periods aimed at reaching expected clinical outcomes.^7,8^

In an attempt to reduce drug resistance, the Centers for Disease Control and Prevention (CDCP) has implemented several strategies in an attempt to reduce antifungal resistance in particular. Strategies include the appropriate prescribing of antifungals in terms of duration and indication, the assessment of antifungals as part of antimicrobial stewardship programmes along with the accurate documentation of dosing, adverse effects, and, lastly, the eventual therapeutic outcome.^9^

Despite fluconazole being one of the most commonly prescribed antifungals, an increase in resistance has been observed in *Candida* spp. This rise in numbers may be because of increased prescription rates, previous exposure and resistant mechanisms,^1,10^ such as genetic alterations. Consequently, this has necessitated the use of alternative treatments such as the use of echinocandins, such as caspo- and anidula-fungin, which inhibit fungal cell wall synthesis. They do this by inhibiting the β-_D_-(1–3)-glucan synthase enzyme to prevent the formation of β-_D_-(1–3)-glucan polymers in the cell wall, and, ultimately, render the cells vulnerable to external stressors.^11^ In addition to proving effective against *Candida* spp., they further possess an attractive safety profile and are associated with minimal drug–drug interactions and adverse effects at therapeutic doses,^12^ particularly in relation to other commonly prescribed antifungals, such as fluconazole.

Despite the aforementioned, a recent South African (SA) report observed multidrug resistance, especially for *C. albicans, C. glabrata* and *C. tropicalis.* This finding indicates a formidable threat to the public-health sector, which could be indicative of the inappropriate prescription and use of antifungal treatments.^1^ While the narrow selection of available treatments complicates *Candida* infections, the situation is exacerbated by these organisms having biofilm activity within indwelling devices and catheters.^13^ This unique characteristic of Candida spp. has led to many clinical guidelines recommending the removal or replacement of indwelling devices after diagnoses of invasive candidiasis or candidemia.^5,14,15^

In 2012, the European Society for Clinical Microbiology and Infectious Diseases (ESCMID), recommended that treatment be simplified to an oral agent after 10 days of intravenous (IV) treatment if an individual patient is stable and able to tolerate an oral form and when the species is susceptible to the treatment.^15^ Furthermore, in its 2016 guidelines, the Infectious Diseases Society of America (IDSA) recommend that clinically stable patients’ treatments be changed from an IV form to an oral agent (i.e. fluconazole) within 5–7 days. In this regard, however, it is imperative that both suspected or confirmed *Candida* spp. be susceptible to at least one of the available oral agents. In addition, individual patients should have negative blood cultures repeatedly.^14^ In general, this approach, namely the changing of IV to oral therapy, is aimed at minimising treatment and hospitalisation periods, among others. In SA, a local Quality Alert was issued by a private hospital group that based its recommendations on the ESCMID guidelines, which pharmacists working for this particular chain were to implement to ensure that echinocandins are appropriately used.

The duration of therapy for patients with the previously mentioned *Candida* infections remains a challenge because of various reasons. These factors include the following^10,14^: firstly, candidemia is associated with a high mortality rate; secondly, there are several limitations related to an early diagnosis; thirdly, the variety of different *Candida* spp. are geographically distributed, each having its own unique challenges; fourthly, some species possess biofilm activity; and, lastly, the *Candida* can be deep-seated within different organs. While these treatment-related challenges have hampered guidelines and the literature in terms of reaching an accurate consensus regarding the recommended length of treatment period, research has established that a 14-day course of IV treatment should be implemented after the first negative blood culture. Consequently, this study sought to determine whether doctors comply with the available guidelines.

## Methods

The study took place within the context of a private hospital in the SA province, of Gauteng. The target population comprised of all patients receiving antifungal treatments from 01 January 2015 to 31 December 2015. Furthermore, in order to take part, patients had to comply with the study’s inclusion criteria. Patients, therefore, had to be adults admitted to a specific private hospital who were receiving antifungal treatment during their hospitalisation. A total of 146 met the criteria and, consequently, took part in the study.

This particular study followed a quantitative research design method and was observational and descriptive in nature. This approach was chosen because the data were retrospective and had no influence on the findings.

Using a data collection form, this study captured the following data fields, namely patients’ demographic information, including age and ward admitted in hospital; type of the IV echinocandin that the patient was initiated on; loading dose (LD); prescribed daily dose (PDD); starting date and end date of echinocandin treatment; and cost of antifungal treatment, including the blood tests and blood cultures performed.

Further inclusion criteria included: (1) all patients 18 years of age and older and (2) the period during which all patients 18 years of age and older were started on IV echinocandin treatment from 01 January 2015 to 31 December 2015. The specific time frame was chosen because anidulafungin only became available at the end of 2014 in SA. Patients were excluded if (1) they were admitted while on any acute or chronic antifungal treatment, (2) pregnant patients and (3) patients whose echinocandin treatment was changed during their hospital stay to some other IV antifungal.

Important variables in this study included the type of antifungal, the dosage of antifungal treatment, the duration of treatment, the de-escalation of therapy, blood results and cultures and the average cost of antifungal treatment.

In consultation with the Statistical Consultation Services of the NWU, the Statistical Analysis System^®^, SAS 9.3^®^ (SAS Institute Inc., 2009) was used to evaluate the data. In this regard, descriptive statistics, such as frequencies (*n*), percentages (%), means, standard deviations (s.ds), and 95% confidence intervals (CIs), were used to express all variables. In addition, the study used Cohen’s *d*-value to define the practical significance of the results (with *d* ≥ 0.8 seen as a large effect with practical significance). To determine the association between two groups, the two-sample *t*-test was used and the association between two categorical variables were determined by the Pearson’s chi-square test. Lastly, Cramér’s *V* was used to determine the practical significance of the results (with *V* ≥ 0.5 accepted as a large effect with practical significance).

## Results

### General prescribing patterns of echinocandins

One hundred and forty-six patient records were retrieved. These were examined to determine echinocandin-related prescription patterns. Consequently, it was found that 102 (69.86%) patients received caspofungin, while a further 44 (30.13%) received anidulafungin. Moreover, of the 102 who received the former, 99 (97.06%) patients received a LD of 70 mg, while 3 (2.90%) patients did not receive a LD. For the 44 patients who received anidulafungin, only 30% (68.18%) received a LD of 200 mg, while 14 (31.82%) either received an inappropriate LD or did not receive a LD at all.

The study also established that 98 (98.1%) received the correct PDD, while 4 (3.92%) received 70 mg per day. In terms of anidulafungin, while the PDD for this specific treatment is 100 mg IV per day, this study determined that only 19 (43.2%) received the correct PDD. One patient (2.3%) received 400 mg, 23 (52.3%) received 200 mg and one (2.3%) received 50 mg daily IV. Table 1 was drawn up to establish whether the echinocandins were prescribed with the appropriate LD.

**TABLE 1:** Frequency and percentage of the loading dose of anidulafungin and caspofungin.

Products	Frequency	Percentage
**Anidulafungin**		
Yes	30	68.2
No	14	31.8
Total	44	-
**Caspofungin**		
Yes	99	97.1
No	3	2.9
Total	102	-

This table indicates that cases of appropriately prescribed LD outnumbered those in which LD was administered for either anidulafungin or caspofungin treatment regimens. In the majority of patients, the collected data further determined that the average dose of anidulafungin was 200 mg IV per day. More specifically, only one patient was prescribed 50 mg IV per day, 19 received 100 mg IV per day and 23 patients were prescribed 200 mg IV per day. Furthermore, only one patient received 400 mg IV per day. As previously stated, for caspofungin, 98 patients received 50 mg IV per day, while only four patients received 70 mg IV per day.

### A comparison of the average duration of treatment of patients with blood cultures and those without

An independent *t*-test and Cohen’s *d* value were used to compare the average treatment duration of patients with blood cultures to those without. The results are presented in Table 2.

**TABLE 2:** A comparison of the average duration of treatment between patients with blood cultures and patients without blood cultures.

Statistical measures	Yes (positive blood culture)	No (blood culture)	Independent *t*-test	*p*-value	Cohen’s *d* value
*N*	129	17	−0.141	0.888	0.031
Mean (s.d.)	9.7 (7.306)	10.0 (8.7)	-		-
Median	8.0	7.0	-		-
95% Confidence interval for mean	8.4; 11.001	5.5; 14.5	-		-

s.d., standard deviation.

Here, of the 146 patients in the study, the mean days on therapy were 9.7, regardless of whether blood cultures were performed.

## Discussion

### International guidelines and general patterns of prescription

As seen in Table 3, three international guidelines and a local Quality Alert by a private SA hospital group were studied to explore the correct LD and PDD for echinocandins (Messina A, 2017, personal communication, July 01).^5,14,15,16^ These guidelines reached a consensus in terms of the dosing of this particular drug. Subsequently, they were used to measure the degree of compliance in the prescribing patterns of the drug; particularly, in adult patients who were privately hospitalised. Within the context of this study setting, we noted that these drugs were, on the whole, correctly prescribed, according to the LD and PDD. As mentioned, the caspofungin-related results were recorded as 98 patients who received daily 50 mg IV and four who received 70 mg IV per day. The literature recommends^1,3,4,5,7,8,14,17^ that the daily dose of this drug should be 50 mg IV daily, while the anidulafungin-related dosage is 100 mg IV per day. The study, therefore, concluded that although most patients were receiving the correct daily dose of caspofungin, the daily dose of anidulafungin was not adhered to.

**TABLE 3:** The different international guidelines and local Quality Alert pertaining the loading dose and prescribing daily dose for the prescribing of echinocandins.

Therapeutic parameter	Drug	IDSA guidelines^14^	ESCMID guidelines^15^	An Italian consensus for invasive candidiasis management (ITALIC)^5^	Australian consensus guidelines for yeast infections^16^	Quality alert in study setting
Loading dose (LD) and maintenance dose (MD)	Caspofungin	Loading dose of 70 mg IV and then 50 mg IV MD daily.	A LD of 70 mg IV and a daily MD of 50 mg IV thereafter for initial targeted treatment of candidemia and invasive candidiasis in adult patients. The dosage of caspofungin should however be increased to 70 mg IV daily in patients with a larger body mass index.	Not discussed	The LD is 70 mg IV for the first 24 h, and then a MD 50 mg IV daily.	An initial LD of 70 mg IV and then a daily MD of 50 mg IV. A LD of 70 mg IV daily is recommended for patients of ≥ 80 kg.
-	Anidulafungin	A LD of 200 mg IV and then 100 mg IV daily as initial therapy for candidemia in non-neutropenic patients.	Initial LD of 200 mg IV and then a daily dosage of 100 mg IV for the maintenance of anti-candidemia and invasive candidiasis in adult patients.	-	Initial LD of 200 mg IV for the first 24 h and then maintaining the dose of 100 mg IV daily.	A LD of 200 mg IV and then 100 mg IV every day as MD.
Duration of treatment	-	For candidemia without metastatic complications is the recommended duration of therapy 2 weeks after the documented cessation of *Candida* spp. from the bloodstream and resolution of symptoms pertaining candidemia (strong recommendation; moderate quality of evidence).	The length of duration of treatment depends on the extent of organ involvement, but ESCMID prefers 14 days of treatment after a patient tests negative for candidemia. A patient tests positive until daily blood cultures are negative.	All patients are treated for at least 14 days after the last positive blood culture.	For deep tissue *Candida*, the treatment with systemic antifungals is prescribed for at least 2 weeks after the last positive sterile site culture.	Continuous therapy for 2 weeks after the first negative blood culture. Daily blood cultures should be taken until candidemia tests negative.

*Source:* Please see the full reference list of the article Scudeller L, Viscoli C, Merichetti F, et al. An Italian consensus for invasive candidiasis management (ITALIC). Infection. 2013;42(2):263 -279. https://doi.org/10.1007/s15010-013-0558-0, for more information

ESCMID, European Society for Clinical Microbiology and Infectious Diseases; IDSA, Infectious Diseases Society of America; IV, intravenous.

### A comparison of the average duration of treatment in patients with blood cultures and those without

Out of the total number of patients (146), 129 were recorded as having positive blood cultures, while only 17 did not have a blood culture at all. Moreover, the mean duration of echinocandin therapy for the former was lower than those without, namely 9.7% and 10.0%, respectively. This mean was considered while measuring the blood cultures’ performances because the literature states that therapy should be ceased after 14 days of IV treatment, after patients’ first negative blood cultures.^5,14,15^ This indicates that those patients with positive blood cultures were administered antifungal therapy for a shorter period than their counterparts without blood cultures.

The s.d. for positive blood culture patients was also determined to be lower than those without. This implies that their values have less variability than the values of those without blood cultures. In this regard, both medians are smaller than the corresponding means, which suggests that there is a positive skew in their distribution. Consequently, the probability density function has a longer tail on the right.

The study further established that the *p*-value of 0.888 was greater than the *p*-value of significance (0.05) meaning that there was no statistically significant difference between the average duration of treatment of patients with blood cultures and those without. Moreover, it can be seen as a large effect when Cohen’s *d* value is ≥ 0.8. In this study, Cohen’s *d* value = 0.031, which indicates that there is a small effect of no practical significance (Messina A, 2017, personal communication, July 01).^5,14,15,16^

In light of the given results, this study has proven important in terms of the relevance of antifungal stewardship and the practice of appropriate dosage.

## Conclusion

From the given results and discussion, it is concluded that prescribing doctors working in this particular setting comply with international guidelines regarding the dosing of echinocandins. Moreover, it was determined that the presence or lack thereof of a blood culture does not greatly impact the duration of the treatment therapy. Consequently, we recommend that more research be conducted to accurately compare guidelines with the actual compliance with the duration period.

Various principles regarding echinocandins have been set according to the international guidelines pertaining to the prescription thereof (Messina A, 2017, personal communication, July 01).^5,14,15,16^ In order to ensure pharmacists use echinocandins appropriately, the local hospital group set up a Quality Alert. This alert was already being implemented in 2012, at least 3 years before the onset of echinocandin-resistant organisms, such as *C. auris*.^1,3,9,17,18^ Subsequent reports have established that this type of resistance is increasingly common, and, therefore, the appropriate use of echinocandins should be promoted with particular attention paid to therapy durations, the correct dosages, and the uses of blood cultures to assist practitioners in de-escalating *Candida* species and the total cost of treatments relating to them.^9,17,18^

This study aimed to investigate the available literature pertaining to the utilisation of echinocandins in comparison with the current patterns of prescription of these drugs within a private-practice setting. The results can be used to develop hospital-specific algorithms for *Candida*-infected patients. In addition, this study highlighted the value of guidelines related to available antifungals and how this implementation may lead to more positive outcomes.

This study had various limitations, namely its outcomes depending on how thoroughly patients’ prescription charts were completed by doctors; the researcher’s lack of clinical data in terms of changing catheters and lastly, the study’s reliance on a random sample because of its inclusion and exclusion criteria. In the light of this, future studies may render more accurate results owing to more correct prescription charts.
